# Involvement of a putative ATP-Binding Cassette (ABC) Involved in manganese transport in virulence of *Listeria monocytogenes*

**DOI:** 10.1371/journal.pone.0268924

**Published:** 2022-05-26

**Authors:** Yanhong Liu, Brian ByongKwon Yoo, Cheng-An Hwang, Mira Rakic Martinez, Atin R. Datta, Pina M. Fratamico

**Affiliations:** 1 Eastern Regional Research Center, Agricultural Research Service, U.S. Department of Agriculture, Wyndmoor, PA, United States of America; 2 Centers for Disease Control and Prevention, Atlanta, GA, United States of America; 3 Center for Biologics Evaluation and Research, U.S. Food and Drug Administration, Silver Spring, MD, United States of America; 4 Center for Food Safety and Applied Nutrition, U.S. Food and Drug Administration, College Park, MD, United States of America; Purdue University, UNITED STATES

## Abstract

*Listeria monocytogenes* is a foodborne pathogen and the causative agent of listeriosis, a disease associated with high fatality (20–30%) and hospitalization rates (>95%). ATP-Binding Cassette (ABC) transporters have been demonstrated to be involved in the general stress response. In previous studies, in-frame deletion mutants of the ABC transporter genes, *LMOf2365_1875* and *LMOf2365_1877*, were constructed and analyzed; however, additional work is needed to investigate the virulence potential of these deletion mutants. In this study, two *in vitro* methods and one *in vivo* model were used to investigate the virulence potential of in-frame deletion mutants of ABC transporter genes. First, the invasion efficiency in host cells was measured using the HT-29 human cell line. Second, cell-to-cell spread activity was measured using a plaque forming assay. Lastly, virulence potential of the mutants was tested in the *Galleria mellonella* wax moth model. Our results demonstrated that the deletion mutant, ⊿*LMOf2365_1875*, displayed decreased invasion and cell-to-cell spread efficiency in comparison to the wild-type, *LMOf2365*, indicating that *LMOf2365_1875* may be required for virulence. Furthermore, the reduced virulence of these mutants was confirmed using the *Galleria mellonella* wax moth model. In addition, the expression levels of 15 virulence and stress-related genes were analyzed by RT-PCR assays using stationary phase cells. Our results showed that virulence-related gene expression levels from the deletion mutants were elevated (15/15 genes from ⊿*LMOf2365_1877* and 7/15 genes from ⊿*LMOf2365_1875*) compared to the wild type *LMOf2365*, suggesting that ABC transporters may negatively regulate virulence gene expression under specific conditions. The expression level of the stress-related gene, *clpE*, also was increased in both deletion mutants, indicating the involvement of ABC transporters in the stress response. Taken together, our findings suggest that ABC transporters may be used as potential targets to develop new therapeutic strategies to control *L*. *monocytogenes*.

## Introduction

*Listeria monocytogenes*, a Gram-positive foodborne pathogen, is an important public health concern since it can cause listeriosis associated with a mortality rate of approximately 20 to 30% in animals and humans [1]. Listeriosis outbreaks have been associated with the consumption of contaminated food products, which include ready-to-eat (RTE) meats and dairy and more recently fresh produce [2–4]. *L*. *monocytogenes* is also commonly found in the environment, and it is difficult to eliminate this pathogen from food processing facilities since it is able to survive under harsh conditions such as low pH and high salt [5].

Listeriosis occurs primarily in immunocompromised individuals, including pregnant women, the newborn, and the elderly [1]. *L*. *monocytogenes* virulence involves adhesion and invasion to host cells, escape from vacuoles, intracellular growth, and cell-to-cell spread [6]. The activity of well-characterized virulence factors is involved in each stage of the process [7], and many genes linked to each stage have been identified [8,9]. The *prfA* gene encodes a transcriptional regulator that turns on transcription of several virulence genes, including *hly*, *plcA*, *plcB*, and *inlA* [10,11]. A transcriptional regulator, encoded by the *sigB* gene positively regulates transcription of stress-related genes, including *clpC* and *clpE* [12]. Several genes involved in adhesion are *actA*, *ami*, *fbpA*, and *flaA*, and internalin A and B (*inlA* and *inlB*) that facilitate invasion into mammalian cells [13]. The *hly* gene encodes for listeriolysin O, a pore-forming toxin, which in combination with the action of two phospholipases, *plcA* and *plcB*, is responsible for escape of *L*. *monocytogenes* from vacuoles. Intracellular motility and cell-to-cell spread involve the action of the *actA* and *iap* genes [6].

In recent years, an infection model using larvae of the greater wax moth, *Galleria mellonella*, has been shown to be a promising model to assess virulence of numerous human pathogens, including *L*. *monocytogenes* [14–16]. Advantages of this model are its low cost, easy manipulation, ethical acceptability, and the capability to incubate larvae at 37°C, which is human body temperature and is a prerequisite for the optimal expression of various key virulence factors in *L*. *monocytogenes* [17]. Most importantly, the innate immune system of *G*. *mellonella* resembles that of mammals, with enzymes, reactive oxygen species, and antimicrobial peptides necessary against protection from bacterial infection [14]. In addition, the *G*. *mellonella* model has also been successfully utilized to explore cadmium resistance in *L*. *monocytogenes* [18], as well as comparison of the transcriptomes from different isolates [19].

ATP-Binding Cassette (ABC) transporters serve as major transport systems in bacteria [20]. More than 30 copies of different ABC transporters are found in the *L*. *monocytogenes* genome [21]. Typically, an ABC transporter consists of several subunits, including a nucleotide-binding domain, a transmembrane domain, and/or a solute-binding domain [22]. ABC transporters can be used as targets in the development of antibacterial vaccines and therapies [23]. In addition to transport, some ABC transporters have been demonstrated to be involved in virulence. For example, an ABC transporter that is associated with resistance to antimicrobial peptides contributes to the virulence of *Salmonella* [24].

Liu and Ream [25] showed that *LMOf2365_1875* (ABC transporter, manganese-binding protein), *LMOf2365_1876* (manganese ABC transporter; permease protein), and *LMOf2365_1877* (manganese ABC transporter; ATP-binding protein) were highly induced in milk at 4°C; however, this ABC transporter operon was inhibited in RTE meats [26]. Magnesium is the potential substrate for this transporter, and it is also present in other *L*. *monocytogenes* strains [27,28]. To our knowledge, it is not under control of key *L*. *monocytogenes* transcriptional regulators such as SigB. Previous studies have shown that the in-frame deletion mutants ⊿*LMOf2365_1875* and ⊿*LMOf2365_1877* had no overall growth defects in Brain Heart Infusion (BHI) medium, but were sensitive to salt, acid, and nisin, indicating that *LMOf2365_1875* and *LMOf2365_1877* may be involved in the general stress response [21]. However, there have been no studies on the virulence potential of these deletion mutants. In this paper, we tested the virulence potential of the two in-frame deletion mutants of ⊿*LMOf2365_1875* and ⊿*LMOf2365_1877* to gain insight into the possible role of the ABC transporter during infection of the human host.

Manganese is involved in bacterial virulence [22,29]. For example, acquisition of Mn (II) is required for intracellular survival and replication of *Salmonella enterica* serovar Typhimurium in macrophages and for virulence *in vivo* [30]. Since *LMOf2365_1875* encodes for a putative ABC transporter, manganese-binding protein, we hypothesized that manganese transport may be blocked in ⊿*LMOf2365_1875*; therefore, virulence was reduced in *L*. *monocytogenes*. While this hypothesis needs further testing, it is supported by the following lines of evidence. Manganese also plays an important role in streptococcal virulence [31]. An ABC transporter named MtsA that is involved in manganese transport in *Streptococcus pyogenes* was related to virulence since a deletion mutant resulted in attenuated virulence [32]. MtsA shares 98% similarity with LMOf2365_1877(AAT04647.1) and 72% similarity with LMOf2365_1875 (AAT04645.1). In addition, an *Agrobacterium tumefaciens* mutant with a manganese transport deficiency had attenuated virulence in plants [33]. Similarly, iron acquisition is also required for virulence in *L*. *monocytogenes* since an ABC transporter mutant impaired in heme uptake displayed decreased virulence [34].

## Materials and methods

### Bacterial strains and cell line culture conditions

*L*. *monocytogenes* strain F2365 (isolated from Mexican-style soft cheese) [35] was used in the current study since its genome is fully sequenced and annotated [28]. *L*. *monocytogenes* F2365, *L*. *monocytogenes* Scott A, *L*. *innocua*, and two isogenic deletion mutants (⊿*LMOf2365_1875 and* ⊿*LMOf2365_1877*) of the parent strain *LMOf*2365 (Table 1) stored at -80°C as glycerol stocks were streaked onto BHI agar (Sigma-Aldrich St. Louis, MO) and incubated at 37°C for overnight prior to performing each experiment. The human colon adenocarcinoma cell line HT-29 (ATCC, Manassas, VA, USA) was maintained as described previously [36].

**Table 1:** *Listeria* strains used in this study.

Bacterial strains	Reference/source
*L*. *monocytogenes* F2365 wild-type serotype 4b strain, genome sequenced	[28]
*L*. *monocytogenes* Scott A	Gift from R. D. Joerger (University of Delaware)
*L*. *innocua*	^a^ERRC collection
*ΔLMOf2365_1875*, 1875deletion	[21]
*Δ LMOf2365_1877*, 1877deletion	[21]

^a^Eastern Regional Research Center.

### Cell invasion assays

HT-29 cells (ATCC HTN-38) were used to determine the virulence of the *Listeria* strains [37]. *L*. *monocytogenes* strains (*L*. *monocytogenes* Scott A, isogenic deletion mutants *ΔLMOf2365_1875* and *ΔLMOf2365_1877*, and the parental *LMOf2365)* and *L*. *innocua* were used for the invasion assays performed as described previously [36]. In brief, HT-29 cells were grown in 24-well tissue culture plates for 5 days to obtain almost confluent monolayers. Strains of *L*. *monocytogenes* (*L*. *monocytogenes* Scott A, isogenic deletion mutants *ΔLMOf2365_1875* and *ΔLMOf2365_1877*, and the parental *LMOf2365)* and *L*. *innocua* were grown to log-phase (OD_600nm_ ~0.3) at 37°C. HT-29 cell monolayers incubated in medium without antibiotics for 24 h were infected for 1 h at 37°C with 10^7^ CFU bacterial cells in 300 μl BHI medium (Becton Dickinson and Co., Sparks, MD). The cell monolayers were washed with DMEM and incubated in DMEM containing gentamicin (100 μg/ml) for 1.5 h at 37°C. HT-29 cell monolayers were gently washed three times with phosphate buffered saline (pH 7.4) and then disrupted with 1 ml cold sterile water (4°C). Viable bacteria were counted after plating serial dilutions onto TSA. The results were expressed as the percentage of CFU recovered after 2 h relative to the number of bacterial cells deposited per well. Three independent experiments were performed for each strain.

### Plaque forming assays (PFAs)

Strains of *L*. *monocytogenes* (*L*. *monocytogenes* Scott A, isogenic deletion mutants *ΔLMOf2365_1875* and *ΔLMOf2365_1877*, and the parental *LMOf2365)* and *L*. *innocua* were used for PFA assays, which were performed using HT-29 cells as described previously [36,37]. In brief, confluent HT-29 cell monolayers were incubated in medium without antibiotics for 24 h. The log-phase *Listeria* cells (described above) were used to infect HT-29 cell monolayers with a dilution series of 10^2^ to 10^7^ CFU/ml cells per well, and then incubated for 2 h at 37°C. After removing the bacterial suspensions, cell monolayers were washed with DMEM and incubated in DMEM containing 100 μg/ml of gentamicin for 1.5 h. Each well was then covered with DMEM with 0.5% agarose containing 10 μg/ml gentamicin. After solidification, 400 μl of the same liquid medium were added to the top of the agar to prevent starvation. Tissue culture plates were incubated for 3 days at 37°C under 5% CO_2_ (v/v). Enumeration of formed plaques was performed using an inverted microscope. The results were expressed as log numbers of plaques per 10^7^ CFU/ml deposited per well. Experiments were performed in duplicate and repeated twice for each strain.

### *G*. *mellonella* injection and mortality assay

The assessment of virulence of *L*. *monocytogenes* strains in this study was conducted using the *G*. *mellonella* larvae model, described in our previous work [16]. *L*. *monocytogenes* strains were grown overnight at 37˚C in BHI broth and on BHI agar plates. The overnight liquid cultures in BHI broth were washed twice and serially diluted with phosphate buffered saline (PBS). Appropriate dilutions were plated onto BHI agar and incubated for 24 h at 37˚C to obtain the CFU count. Colony counts were used to calculate the bacterial inoculum for *Galleria* infection. A set of 20 *Galleria* larvae of the similar size (approximately 200–300 mg), light-colored, with a good motility, were inoculated with appropriate dilutions of *L*. *monocytogenes* in the PBS (Fisher BioReagents), for final concentrations of 10^6^ and 10^5^CFU/larva. Inoculated larvae were incubated at 37°C and monitored for mortality and phenotypic changes, including changes in color, motility, dryness, and pupation for a period of seven days. For each treatment, the number of dead larvae was recorded daily for up to seven days. From these data, percent mortality was calculated. Each trial included one set of ten uninoculated larvae and one set of ten larvae inoculated with sterile 0.85% saline solution. One group of uninoculated larvae served as a control for adaptation of *Galleria* larvae to 37°C, while a second group served as a “manipulation” control. Experiments were conducted with three independent trials.

### RT-PCR analysis of virulence and stress-related genes

Strains of *L*. *monocytogenes* (deletion mutants *ΔLMOf2365_1875* and *ΔLMOf2365_1877*, as well as the wild type *LMOf2365* parent strain) (Table 1) were inoculated in 5 ml of BHI and grown with agitation (200 rpm) for 12 h at 37°C. Total RNA was isolated from the above strains of *L*. *monocytogenes* as previously described [25]. Primers targeting 15 genes related to virulence and stress response (Table 2) were designed as previously described [36]. The *spoG* housekeeping gene was used as an internal control (Table 2). cDNA synthesis and real-time PCR analysis conducted in this study were described in our previous work [25]. Reactions without reverse transcriptase were used as negative controls. RT-PCR assays were performed three times for each strain.

**Table 2:** Oligonucleotides used for real-time PCR to evaluate the virulence and stress related genes in *L*. *monocytogenes*.

GENE	Forward primer sequences (5’-3’)	Role in virulence	Product size (bp)
*actA*	F: AAGAGTTGAACGGAGAGGT R: TCAGCTAGGCGATCAATTTC	Adhesion, invasion, vaculole lysis, and intracellular motility [6]	121
*ami*	F: GTAACCATTCGCGATGACTC R: CTTGAATAGCGAACCCTTGA	Adhesion [6]	100
*clpC*	F: GTAACCATTCGCGATGACTC R: CTTGAATAGCGAACCCTTGA	stress response [38]	100
*clpE*	F: CAGAAGCACTAACAGCAGCA R: TCACCGTATTTTCGTCCAGT	stress response [39]	141
*fbpA*	F: GCGGTCGAAGTAGTGAAAGA R: AGCTAGTTCTTGGCGGATTT	adhesion [6]	126
*flaA*	F: CGCAAGAACGTTTAGCATCT R: ATGGATGAGTTTTTGCTTGC	adhesion [40]	127
*hly*	F: GAATGCAATTTCGAGCCTAA R: AGTCATTCCTGGCAAATCAA	Lysis of vacuoles [41]	133
*iap*	F: GAAAAACAAGCTGCACCAGT R: CTGTTGGTGCTTTAGGTGCT	Invasion and actin-based activity [42]	109
*inlA*	F: ATGGGATTTTGCGACAGATA R: CGGAAGGTGGTGTAGTGTTC	Invasion [6]	143
*inlB*	F: ACCTAAACCTCCGACCAAAC R: TCGTTTCCGCTTTAAACATC	Invasion [6]	140
*lap*	F: ATCCCTTCCCTAACACTTGG R: GTGGAAGTTTGAACCATTGC	Adhesion [43]	133
*plcA*	F: AAGACGAGCAAAACAGCAAC R: CTCGTGTCAGTTCTGGGAGT	Vacuole lysis [6]	100
*plcB*	F: ATCCTATCCACCAGGCTACC R: TCTTTCACGTCATTTGAGCA	Vacuole lysis [6]	117
*pfrA*	F: CGCAAGAACGTTTAGCATCT R: ATGGATGAGTTTTTGCTTGC	Transcriptional regulator, virulence [12]	127
*sigB*	F: TCGCAAATATTCCCAAGGTA R: TGACGGTGAATTCCGTGATA	Transcription factor, stress response [12]	127
*spoG*	F: TGACGGTGAATTCCGTGATA R: TCAGCAGAAACGGATTCAGA	Internal control gene [25]	147

### Statistical analysis

Data collected from this study were analyzed using the Student’s t test of the Statistical Analysis Software (SAS Institute Inc., Cary, NC) for paired comparison with P < 0.05 considered significant.

## Results and discussion

### Deletion mutant ⊿*LMOf2365_1875* displayed reduced invasion and cell-to-cell spread activities in the HT-29 cell line

**Fig 1:**
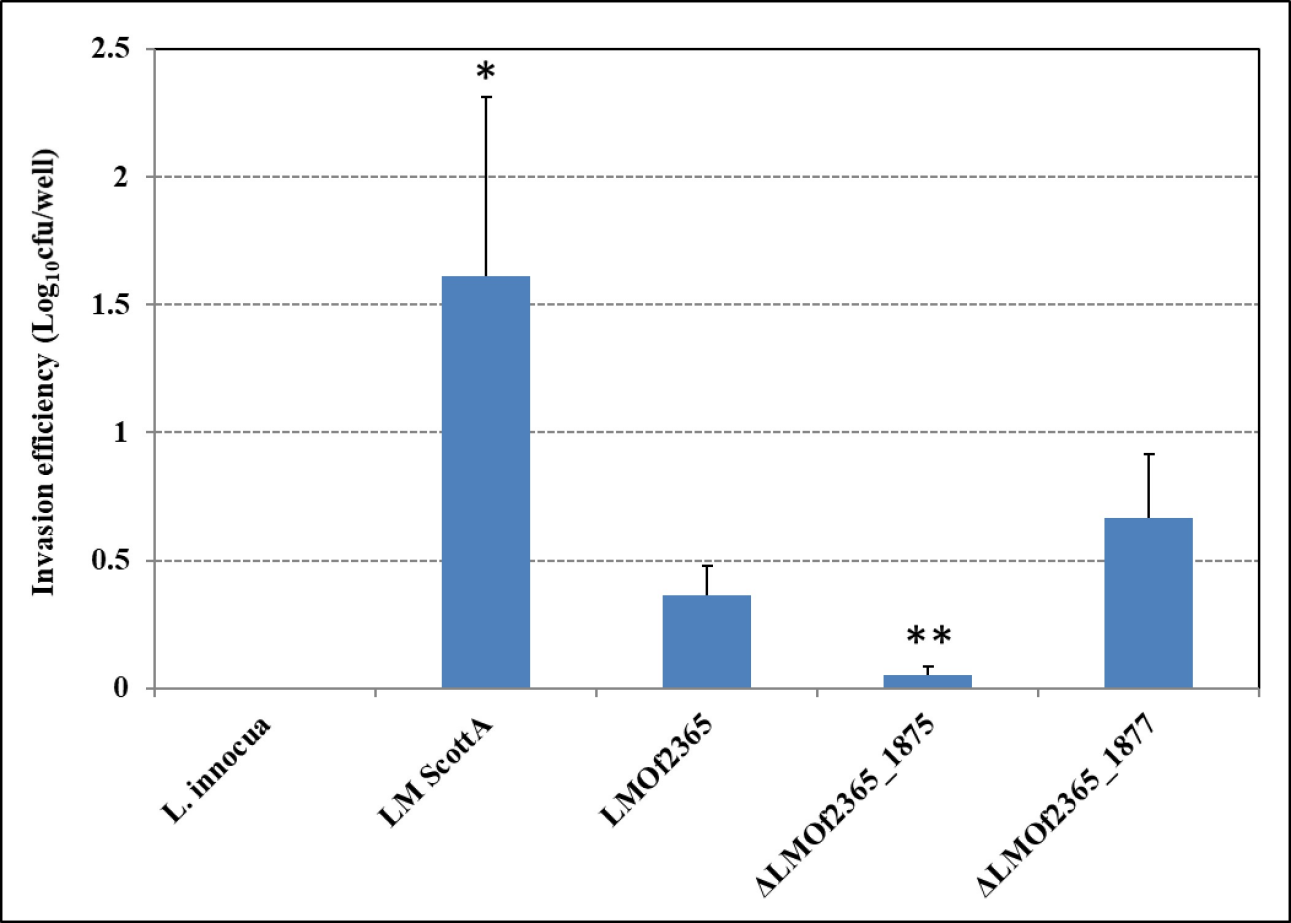
Invasion of *Listeria* strains in HT-29 cells. HT-29 cell monolayers were incubated with *Listeria* strains grown to stationary phase for invasion assays. Viable intracellular bacteria were counted after plating serial dilutions on BHI agar plates. The results were expressed as log numbers of CFU recovered relative to the number of bacterial cells (10^7^) deposited per well. Each experiment was conducted in duplicate and repeated three times. Significant differences from parental wild type *LMOf2365* strain are shown (*, p-value < 0.05; **, p-value < 0.01).

Previous studies identified mutants of an ABC transporter responsible for oligopeptide transport in *L*. *monocytogenes* that were defective in host infection [44]. Since ABC transporters are membrane proteins, they may be involved in the adhesion of *L*. *monocytogenes* to human host cells. To understand if *LMOf2365_1875* and *LMOf2365_1877* are involved in causing host infection, cell invasion and plaque forming *in vitro* assays using HT-29 cell monolayers were employed to test the virulence potential of each deletion mutant. As shown in Fig 1, *L*. *monocytogenes* Scott A expressed the highest invasion (1.6 log_10_ CFU/well), and *L*. *monocytogenes* F2365 *(LMOf2365)* also had a high invasion efficiency (0.4 log_10_ CFU/well). Both *L*. *monocytogenes* F2365 and *L*. *monocytogenes* Scott A belong to serotype 4b strains, which is the serotype most often associated with outbreaks of listeriosis. The adhesion and invasion efficiency *of LMOf2365* was lower compared to the LM Scott A strain (with a p value of 0.03), which is consistent with the fact that *LMOf*2365 has a truncated *inlB* gene that is involved in adhesion and invasion [45]. Non-pathogenic *L*. *innocua*, used as a negative control, showed no invasion. The deletion mutant strain ⊿*LMOf2365_1875* showed a deficiency in invasion (0.1 log_10_ CFU/well) (with a p-value of 0.003), whereas ⊿*LMOf2365_1877* had a slightly higher invasion efficiency (0.7 log_10_ CFU/well) (with a p-value of 0.12) compared to the wild type strain (*LMOf2365*).

**Fig 2:**
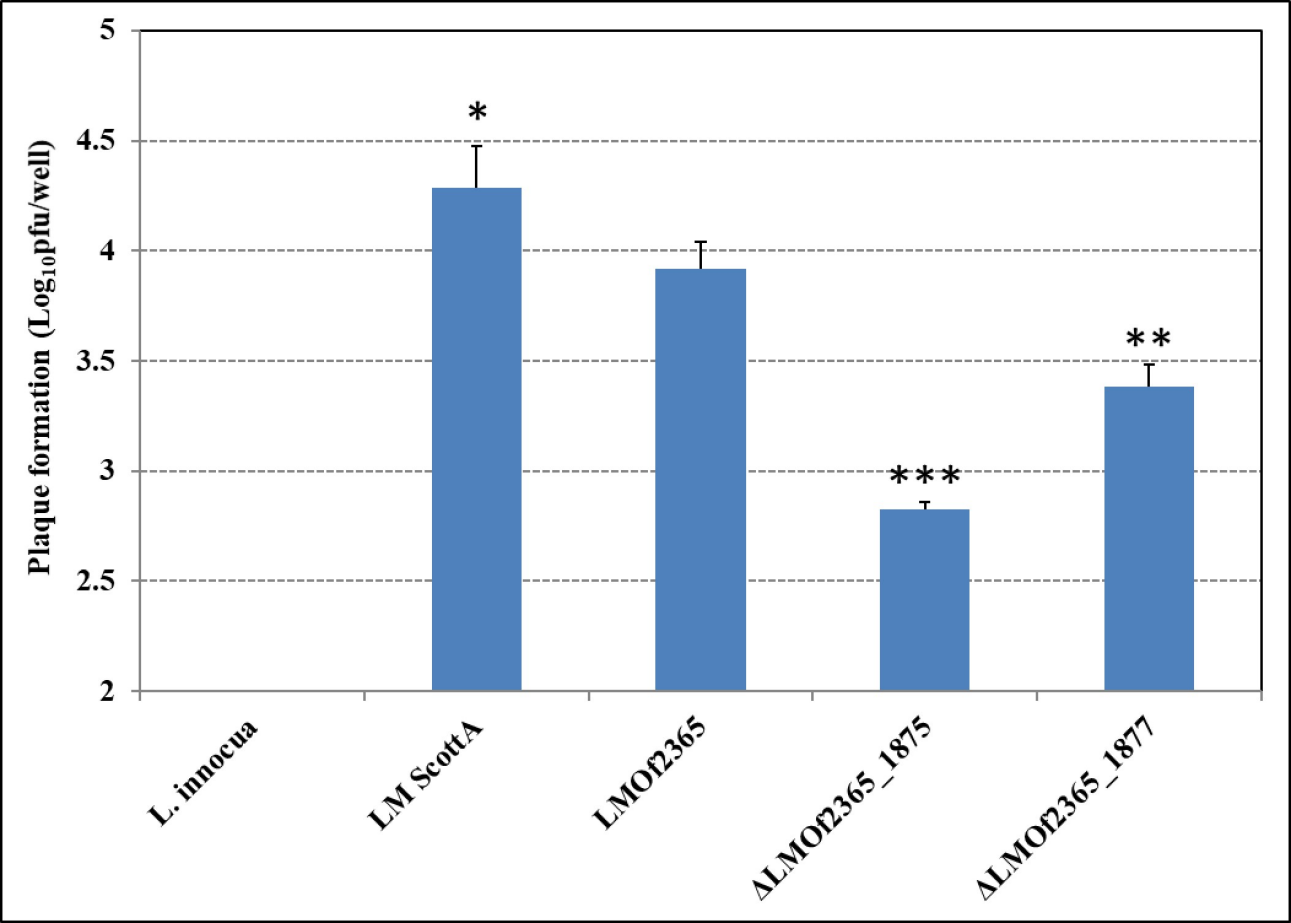
Plaque formation of *Listeria* strains in HT-29 cells. Results are presented as the log_10_ numbers of plaque-forming units (pfu) per well ± standard deviation of triplicate. Significant differences from parental wild type *LMOf2365* strain are shown (*, p-value < 0.05; **, p-value < 0.01; ***, p-value < 0.001).

The other *in vitro* assay for *Listeria* virulence was based on the ability of strains to form plaques on HT-29 monolayers. *L*. *monocytogenes* F2365 formed a higher number of plaques (approximately 3.9 log_10_ pfu/well) in comparison to the two mutants but a lower number compared to Scott A (with a p-value of 0.05) (Fig 2). In contrast, no plaques were visible with the non-pathogenic *L*. *innocua* strain. ⊿*LMOf2365_1875* formed 71% lower number of plaques (2.8 log_10_ pfu/well) compared to the wild type (with a p-value of 0.0002) whereas there was a smaller difference in plaque forming ability between ⊿*LMOf2365_1877* (3.4 log_10_ pfu/well) and the wild type *LMOf2365* (3.9 log_10_ pfu/well) (with a p-value of 0.002). Examining all of the data, results from the invasion and plaque forming virulence assays demonstrated that the deletion mutant ⊿*LMOf2365_1875* displayed some weakness in invasion and intracellular cell-to-cell spread in HT-29 monolayers, suggesting that *LMOf2365_1875* may be required for *L*. *monocytogenes* virulence.

## The ⊿*LMOf2365_1875 and* ⊿*LMOf_1877* showed reduced virulence in the *G*. *mellonella* model

**Fig 3:**
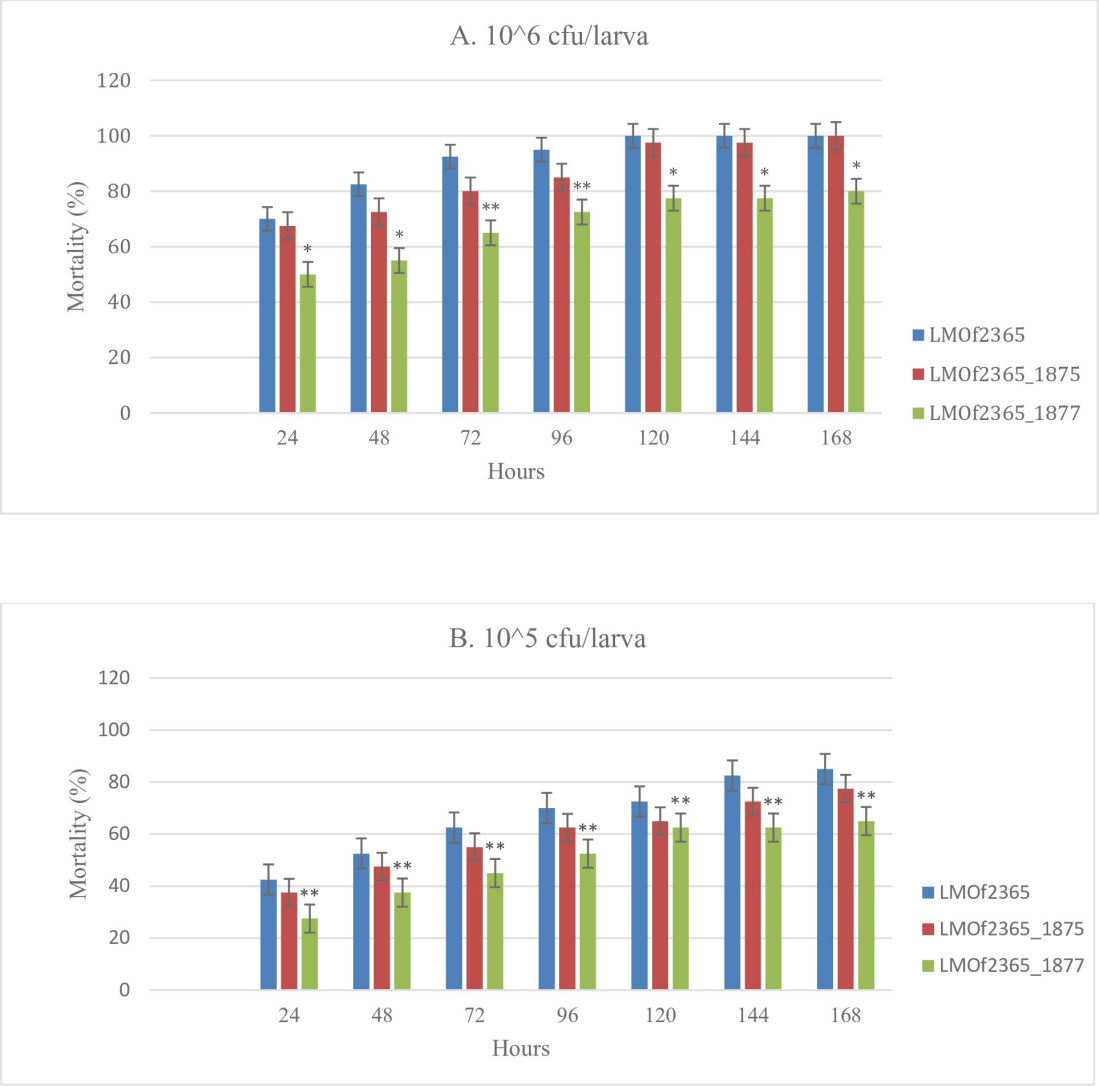
Comparison of mortality rates of deletion mutants ⊿*LMOf2365_1875* and *⊿LMOf2365_1875* with wild type *LMOf2365 in G*. *mellonella* using 10^6^ CFU/larva (A) and 10^5^ CFU/larva (B). *Significant difference from both parental strain and *ΔLMO2365_*1875. ** Significant difference from parental strain. Data presented are the averages of three independent experiments.

The *Galleria mellonella* insect larvae model has been successfully utilized to assess virulence properties of various *L*. *monocytogenes* isogeneic mutants [16]. In this work we studied two isogenic deletion mutants, ⊿*LMOf2365_1875* and ⊿*LMOf2365_1877* and compared them with the parent strain *LMOf2365*. Our results (Fig 3A and 3B) showed that both mutant strains, regardless of the inoculated dose (10^6^ or 10^5^ CFU/larva), exhibited reduced mortality, hence lower virulence potential, compared to the parent strain *LMOf2365*. At the dose of 10^6^ CFU/larva (Fig 3A), virulence of the deletion mutant⊿*LMOf2365_1875* was lower than that of the *LMOf2365* parental strain over the first 96 h of the monitoring period, after which the virulence potential of both strains appeared to be similar. The difference in the first 96 h was not statistically significant. On the other hand, the deletion mutant ⊿*LMOf_1877*, expressed a significantly lower mortality rate/virulence potential compared to both *LMOf2365* and ⊿*LMOf_1875* throughout the monitoring period of infected larvae. We have observed a similar trend at the dose of 10^5^ CFU/larva (Fig 3B). Although the difference in mortality rates between the parental strain and ⊿*LMOf2365_1875*, as well as the mutants themselves, was not significant, the difference between the parent strain *LMOf2365* and *LMOf2365_1877* was significant (Fig 3B). It appears that the mutant *LMOf2365_1877* had an increased effect on virulence compared to ⊿*LMOf2365_1875* as evidenced by lower mortality. This effect is especially expressed at the higher inoculum dose. Previous work [16] showed that *L*. *monocytogenes* isogenic mutants of *prf*A, *hly*A, *vir*R and *vir*S had a significant effect on mortality in the *Galleria* model while *inl*A and *inl*B had marginal effects, which correlates with the known effects of these genes on *Listeria* virulence.

### The expression levels of virulence and stress-related genes were elevated in ⊿*LMOf2365_1875* and ⊿*LMOf2365_1877* under stationary phase

To determine the gene expression levels in ⊿*LMOf2365_1875* and ⊿*LMOf2365_1877* under stationary-phase conditions, 15 genes related to virulence and stress response [46] were chosen for real-time PCR assays. All of the virulence-related genes were up-regulated in ⊿*LMOf2365_1877* in comparison to the wild type parental *LMOf2365* strain (Table 3), indicating that *LMOf2365_1877* negatively regulated virulence and stress gene expression under stationary phase growth conditions. Although the expression level of *pfrA*, the major virulence regulator in *L*. *monocytogenes*, was relatively higher (21.4-fold) in ⊿*LMOf2365_1875* compared to the wild type parental strain *LMOf2365*, expression levels of the genes (*actA*, *plcA*, *plcB* and *hly*) regulated by *pfrA* were not up-regulated in ⊿*LMOf2365_1875*. On the other hand, the expression levels of other virulence-related genes *(ami*, *inlA*, *inlB and fbpA)* were up-regulated. Our previous studies indicated that stationary phase cells of the deletion mutants (⊿*LMOf2365_1875* and ⊿*LMOf2365_1875)* were more resistant to multiple stress conditions [21], indicating that they may contribute to the general stress response. The gene expression levels of three stress-related genes (*sigB*, *clpC*, and *clpE*) were tested using RT-PCR assays. As shown in Table 3, the expression levels of *clpC* were moderately elevated (6.8 and 10.1-fold) in ⊿*LMOf2365_1875* and ⊿*LMOf2365_1877*, respectively. The increased levels of stress-related gene, *clpC*, expression confirmed our previous observation that these deletion mutants may contribute to general stress. In addition, the expression levels of *clpE* and *sigB* were also elevated (6.8 and 4.0-fold, respectively) in ⊿*LMOf2365_1877*.

**Table 3:** Relative expression levels of virulence and stress-related genes in wild-type and deletion mutants of *L*. *monocytogenes* F2365.

	*actA*	*ami*	*iap*	*inlA*	*inlB*	*lap*	*fbpA*	*plcA*	*hly*	*pfrA*	*clpC*	*clpE*	*sigB flaA plcB*
LMOf2365^a^	1	1	1	1	1	1	1	1	1	1	1	1	1 1 1
ΔLMOf2365_1875	-1.4^b^	19.6	1.2	18.3	11.2	-1.0	5.6	1.1	-3.2	21.4	6.9	1.1	-1.4 63.3 –1.4
Δ LMOf2365_1877	8	25.6	10.6	30.7	11.8	4.0	12.3	8.0	7.7	1.9	10.1	6.8	4.0 3.5 12.7

^a^The expression levels of the genes in the mutant strains were normalized to that in wild-type *LMOf2365* strains.

^b^ Numbers are average values from three independent experiments.

In this study, the deletion mutants showed reduced virulence in terms of invasion and cell-to-cell spreading ability; however, a number of virulence genes showed increase expression under stationary-phase growth. This seems to be contradictory, but the gene expression experiments were not performed under conditions that would occur during infection. It is likely that the virulence gene expression levels were repressed due to catabolite repression under stationary-phase growth conditions; however, these genes were de-repressed in ⊿*LMOf2365_1875* and ⊿*LMOf2365_1877*.

We did not perform the complementation experiments for the deleted genes because these deletions are in frame (21), and which by design assures non-interference of other genes at the transcription level. A complementation experiment may not provide any additional information. In addition, gene complementation in *Listeria*, whether by plasmid or by an integration vector, do not exactly mimic the wild type situation because of the difference in genetic machinery involved in complementation and the topology of the complemented gene.

## Conclusions

The virulence potential of the deletion mutants, *ΔLMOf2365_1875* and *ΔLMOf2365_1877*, was assessed using both *in vitro* (invasion and plaque forming ability) and *in vivo* (*G*. *mellonella* insect model) assays. Our study showed for the first time that *LMOf2365_1875* encoding for a manganese-binding protein of an ABC transporter might be required for virulence. In the *G*. *mellonella* model, decreased mortality of the deletion mutant ⊿*LMOf2365_1877* also indicates a possible role in the virulence potential of *L*. *monocytogenes*. In addition, the gene expression levels of *L*. *monocytogenes* virulence and stress-related genes were elevated in *ΔLMOf2365_1877* under normal laboratory growth conditions. Targeting virulence factors could be a promising approach to develop new strategies against resistant microorganisms.
